# Sevoflurane attenuates myocardial ischemia/reperfusion injury by up-regulating microRNA-99a and down-regulating BRD4

**DOI:** 10.1590/acb383123

**Published:** 2023-10-23

**Authors:** Xiaomin Bie, Jiying Ao, Degang Zhu

**Affiliations:** 1 Wuhan No 1 Hospital – Department of Anesthesiology – Wuhan (Hubei) – China.

**Keywords:** Myocardial Ischemia, Reperfusion Injury, Sevoflurane, Bromodomain Containing Proteins, Myocytes, Cardiac

## Abstract

**Purpose:**

It has been explored that sevoflurane (Sevo) is cardioprotective in myocardial ischemia/reperfusion injury (MI/RI) and mediates microRNA (miRNA) expression that control various physiological systems. Enlightened by that, the work was programmed to decode the mechanism of Sevo and miR-99a with the participation of bromodomain-containing protein 4 (BRD4).

**Methods:**

MI/RImodel was established on mice. MI/RI modeled mice were exposed to Sevo or injected with miR-99a or BRD4-related vectors to identify their functions in cardiac function, pathological injury, cardiomyocyte apoptosis, inflammation, and oxidative stress in MI/RI mice. MiR-99a and BRD4 expression in myocardial tissues were tested, and their relation was further validated.

**Results:**

MiR-99a was down-regulated, and BRD4 was up-regulated in MI/RI mice. Sevo up-regulated miR-99a to inhibit BRD4 expression in myocardial tissues of MI/RI mice. Sevo improved cardiac function, relieved myocardial injury, repressed cardiomyocyte apoptosis, and alleviated inflammation and oxidative stress in mice with MI/RI. MiR-99a restoration further enhanced the positive effects of Sevo on mice with MI/RI. Overexpression of BRD4 reversed up-regulation of miR-99a-induced attenuation of MI/RI in mice.

**Conclusions:**

The work delineated that Sevo up-regulates miR-99a to attenuate MI/RI by inhibiting BRD4.

## Introduction

Ischemia/reperfusion injury (I/RI) can results in high morbidity-and-mortality diseases including ischemic stroke and myocardial infarction (MI)^1^. MI is a leading reason of morbidity and mortality around the world, which is the irreversible death of cardiomyocyte due to prolonged hypoxia or fresh blood supply^2^,^3^. Specifically, myocardial I/RI (MI/RI) is the process of restoring blood flow to the ischemic area to rescue myocardial tissues, causing further damage to the heart^4^. The involved mechanism of MI/RI mainly consists of enhanced oxidative stress, inflammation, calcium overload, apoptosis, and mitochondrial injury^5^. Thrombolytic therapy and revascularization can completely reocclude the epicardial coronary artery in patients suffered from myocardial infarction^6^. Given the threatening complications and consequences of MI/RI, searching for potential targets for MI/RI treatment is a priority.

Anesthesia pre-condition is beneficial to reductions in myocardial enzymes, infarct size, and enhancement in cardiac function recovery, therefore reducing 50% myocardium injury^7^. Sevoflurane (Sevo) is one of the inhaled volatile anesthetic agents, and it has been reported to be significant in attenuating MI/RI by restoring the HIF-1/BNIP3-regulated mitochondrial autophagy in GK rats^8^. Lately, it has been revealed that Sevo pre-condition facilitates diabetic MI/RI via differential modulation of p38 and ERK^9^. Also, the protective effects of Sevo have also been manifested in alleviating reperfusion arrhythmia induced by MI/RI by ameliorating TDR and MAPD90^10^.

MicroRNAs (miRNAs) have been suggested in the network of MI/RI, with the involvement of Sevo. For instance, there is a study illustrating that miR-370 contributes to alleviate MI/RI following Sevo preconditioning via the PLIN5-dependent PPAR pathway^11^. Additionally, miR-374 serves as a protector in MI/RI mice pre-conditioned with Sevo through activating the PI3K/Akt pathway^12^. Currently, a study has evidenced that miR-99a exerts functional role in cardiomyocyte oxidative injury and can be developed to treat cardiovascular diseases^13^.

Caught by a research recently, it is disclosed that miR-99a is down-regulated in patients with acute myocardial infarction (AMI), which implies a diagnostic application in AMI^14^. Similarly, miR-99a up-regulation can improve heart remodeling and cardiac function in MI^15^. Bromodomain and extraterminal domain-containing proteins (BETs), including bromodomain-containing protein 4 (BRD4), can accelerate transcription of pro-inflammatory genes and BET suppression offsets inflammation in coronary artery disease^16^. Specifically, BRD4 knockdown can repress cardiomyocyte apoptosis in rats with MI^17^. Although the independent functions of Sevo, miR-99a, and BRD4 in MI/RI have been explored, the integrity of these factors has not been comprehended thoroughly. Therein, the work was programmed to interpret the functions of the Sevo/miR-99a/BRD4 axis in MI/RI.

## Methods

### Ethics statement

Experiments conducted in this study were approved and reviewed by the Animal Ethics Association of our hospital.

### Experimental animals

Male C57 mice, aged 6-8 weeks old and weighing 25-30 g, were supplied by the animal center of our hospital (Hubei, China). Mice were adaptively reared for one week in animal rooms at 24 ± 2°C, with humidity of 50% and 12-h light/dark cycles.

### Myocardial ischemia/reperfusion injury modeling and treatment of mice

Anesthetized by intraperitoneal injection of 2% pentobarbital sodium (50 mg/mL; Sinopharm Group Chemical Reagent Co., Ltd., Shanghai, China), mice were connected to an electrocardiogram to monitor electrode. Then, they were ventilated with an animal ventilator, at respiratory rate of 60 times/min and tidal volume of 13-15 mL. The left fourth intercostal space was opened, and a 6-0 non-invasive absorbable suture was inserted into the left anterior descending coronary artery. The raised ST segment represented the successful coronary occlusion. After 30 min, the suture was released for blood flow restoration. Then, decreased ST segment indicated successful reperfusion. After another 2-h continuous reperfusion, the MI/RI model was successfully established.

Mice were evenly distributed into nine groups (15 each group):

- I/R group;
- Sevo group: after ischemia for 30 min, the mice inhaled 2.8% Sevo (Abbott, IL, USA) for 2 min before reperfusion, continued for 5 min, followed by reperfusion for 120 min);
- Sevo + mimic NC group;
- Sevo + miR-99a mimic group;
- Sevo + inhibitor NC group;
- Sevo + miR-99a inhibitor group;
- Sevo + miR-99a mimic + overexpression (oe)-NC group;
- Sevo + miR-99a mimic + oe-BRD4 group: 24 h before modeling, the related sequence of miR-99a and BRD4 (0.2 μL/g, GenePharma, Shanghai, China) was injected slowly into myocardium (in five different sites in the infarct zone). The thoracic cavity was closed after injection, and mice were given buprenorphine (0.1 mg/kg body weight) for analgesia, and then were treated as the Sevo group;
- A sham group was set as NC, in which normal mice were only treated with suture insertion into the left anterior descending coronary artery.

### Cardiac function-related index detection

At 24-h post MI/RI modeling, the mice were anesthetized once again and connected to a small animal ultrasonic apparatus (VisualSonics, Toronto, Canada) to detect left ventricular ejection fraction (LVEF) and left ventricular fractional shortening (LVFS) along the short axis.

### Enzyme-linked immunosorbent assay

At 24-h post MI/RI modeling, blood samples were collected from orbits of mice. The blood samples were centrifuged at 3,500 rpm to collect the supernatant creatine kinase-myocardial bound (CK-MB). Myoglobin (Mb), cardiac troponin I (cTnI), interleukin (IL)-6, IL-1β, and tumor necrosis factor-α (TNF-α) contents in serum were tested on the basis of enzyme-linked immunosorbent assay (ELISA) detection kits (Shanghai Enzyme-linked Biotechnology Co., Ltd, Shanghai, China). Serum samples were reacted in the reaction wells for 45 min and incubated with biotin-labeled antibody for 30 min. Subsequently, the samples were incubated with horseradish peroxidase-labeled streptavidin for 30 min, developed and added with stopping solution to terminate reaction.

### Hematoxylin-eosin staining

After 24 h of MI/RI modeling, mice from each group were euthanized to obtain the hearts. A portion of the myocardial tissues were fixed in 40 g/L paraformaldehyde, and another part was retained at -80°C for further use. Next, the myocardial tissues were embedded in paraffin and sectioned into 5 μm. The sections were dewaxed by xylene, and stained by hematoxylin-eosin (HE) solution, which was followed by dehydration by gradient ethanol and sealing. Pathological and morphological changes of myocardium were observed under a microscope (Nikon, Tokyo, Japan).

### Transferase-mediated deoxyuridine triphosphate-biotin nick end labeling staining

Myocardial tissues were sectioned into 5 μm, and treated with xylene, gradient ethanol, and distilled water in succession. Following the instructions of transferase-mediated deoxyuridine triphosphate-biotin nick end labeling (TUNEL) in situ apoptosis detection kit (Sangon Biotech Co., Ltd., Shanghai, China), the sections were dropped with biotin labeling solution and diaminobenzidine color developing solution. Lastly, the sections were scanned by the 3DHISTECH Panoramic SCAN system (3DHISTECH, Budapest, Hungary). Observed under five non-overlapping fields, the apoptotic and total nucleus were counted by Image J software: cardiomyocyte apoptosis rate = apoptotic nucleus/total nucleus × 100%.

### Oxidative stress injury-related index detection

Immersed in normal saline to remove blood, myocardial tissues were decomposed to obtain cardiomyocytes^18^. Superoxide dismutase (SOD), glutathione peroxidase (GSH-Px), and GSH kits were applied to detect SOD and GSH-Px activities and GSH content (Beyotime Institute of Biotechnology, Shanghai, China).

### Reverse transcription quantitative polymerase chain reaction

Total RNA from myocardial tissues were collected for reverse transcription quantitative polymerase chain reaction (RT-qPCR). RNA was utilized as a template. U6 was an internal control for miR-99a, while glyceraldehyde-3-phosphate dehydrogenase (GAPDH) for other genes. MiR-99a and BRD4 expression were calculated by the 2^-ΔΔCt^ method. The primer sequences were presented in Suppl. Table 1. The specific steps were as described in the existed study^19^.

### Western blot assay

With reference to the performance in a former study^20^, Western blot assay was implemented. Myocardial tissues were utilized to extract total protein, which was treated with separation by sodium dodecyl sulphate polyacrylamide gel electrophoresis, followed by transferring to polyvinylidene fluoride membrane and sealing in 5% skimmed milk powder. The primary antibodies BRD4 and GAPDH (Abcam, MA, United States of America) were utilized for membrane incubation. The protein was photographed using the Bio-Rad image analysis system (Bio-Rad, CA, United States of America) and analyzed by Quantity One v4.6.2 software.

### Dual luciferase reporter gene assay

Gene prediction software (https://cm.jefferson.edu/rna22/Precomputed/) was adopted to predict the target gene ofmiR-99a. The 3’UTR fragments of BRD4 containing both mutant (MUT) and wild-type (WT) binding sites of miR-99a were amplified by polymerase chain reation (PCR) and cloned into the vector pMIR-REPORLuciferase (Promega, Madison, WI, United States of America) to form luciferase reporter vectors. HEK-293T cells (Invitrogen Life Technologies, Carlsbad, CA, United States of America) were spread on 96-well plates at 4 × 10^3^ cells/well. HEK-293T cells were transfected withmiR-99a mimic + BRD4-wild type (WT), miR-99a mimic + BRD4-mutant type (MUT), mimic NC + BRD4-WT, or mimic NC + BRD4-MUT with lipofectamine 2000 (Invitrogen). At 48 h after transfection, cells were harvested, and luciferase activity was measured using dual luciferase reporter gene kit (Promega, Madison, WI, United States of America). The ratio of firefly luciferase activity to Renilla luciferase activity indicated the relative activity of luciferase^21^.

### Statistical analysis

All data were processed by Statistical Package for the Social Sciences (SPSS) 21.0 statistical software (IBM, Armonk, NY, United States of America). Measurement data were expressed as mean ± standard deviation. During the statistical analysis of the data, a normality test on the data were conducted, and it was found that it was consistent with the normality. Therefore, we used independent sample t-test to compare the differences between the two groups. Multi-group differences were analyzed by one-way analysis of variance (ANOVA) combined with Tukey’s multiple comparison test. *P* < 0.05 represented statistical value.

## Results

### Sevoflurane improves myocardial ischemia/reperfusion injury in mice

**Figure 1:**
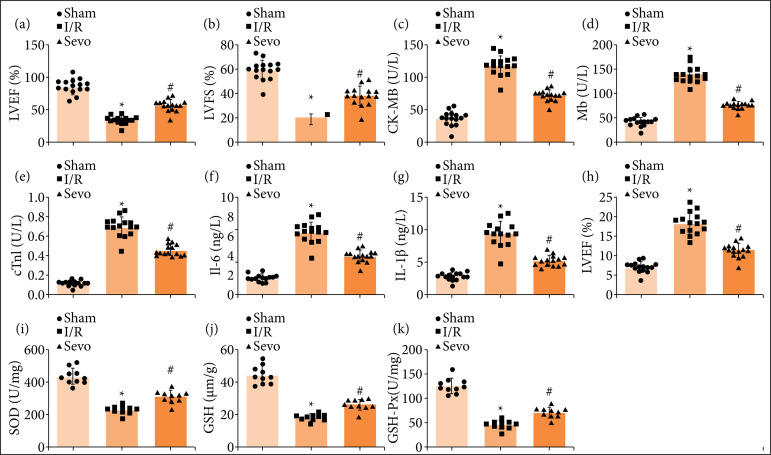
Sevo improves MI/RI in mice. (**a** and **b**) LVEF and LVFS of mice in each group (n = 15); (**c–e**) CK-MB, Mb, and cTnI contents in serum of mice in each group (n = 15); (f–h) IL-6, IL-1β, and TNF-α contents in serum of mice in each group (n = 15); (**i–k**) SOD, GSH, and GSH-Px levels of mice in each group (n = 10).

The protective role of Sevo in MI/RI has been documented in advance^12^,^21^,^22^. A mouse MI/RI model was established, and 2.8% Sevo was inhaled to treat mice after 30 min of ischemia. Echocardiographic examination of cardiac function pointed out that MI/RI mice impaired cardiac function, which was reflected by decreased LVEF and LVFS values (Figs. 1a and 1b). ELISA was implemented for testing myocardial injury markers CK-MB, Mb, and cTnI expression levels in serum, and the findings suggested that CK-MB, Mb, and cTnI expression levels were heightened in MI/RI mice (Figs. 1c–1e). IL-6, IL-1β, and TNF-α contents were all typical inflammatory factors in MI/RI, and their expression levels reflected the inflammation level of MI/RI. Meanwhile, oxidative stress was crucial in the pathological progress of MI/RI. Detected by ELISA and oxidative stress detection kits, IL-6, IL-1β, and TNF-α contents were raised, while SOD and GSH-Px activities and GSH content were decreased in MI/RI mice (Figs. 1f–1k).

I/RI: ischemia/reperfusion injury; MI/RI: myocardial ischemia/reperfusion injury; Sevo: sevoflurane; LVEF: left ventricular ejection fraction; LVFS: left ventricular fractional shortening; CK-MB: creatine kinase-myocardial bound; Mb: myoglobin; cTnI: cardiac troponin I; IL: interleukin; TNF-α: tumor necrosis factor-α; SOD: superoxide dismutase; GSH: glutathione; GSH-Px: glutathione peroxidase; *P < 0.05 compared with the sham group; #*P* < 0.05 compared with the I/R group; @measurement data were expressed as mean ± standard deviation. One-way analysis of variance was utilized for discrepancy among groups, followed by Tukey’s multiple comparisons test.

**Figure 2:**
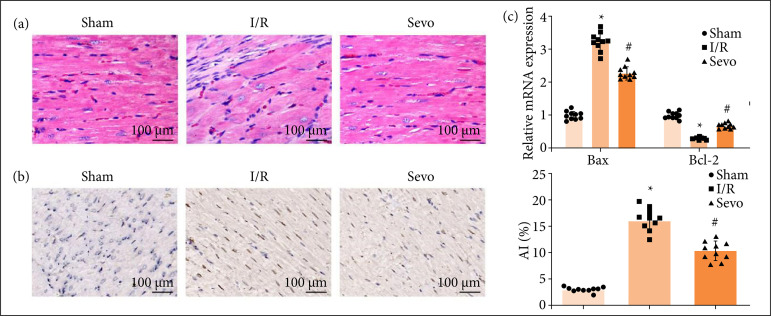
Sevo improves MI/RI in mice. **(a)** Representative myocardial tissues stained by HE solution (n = 10); **(b)** representative myocardial tissues stained by TUNEL staining solution (n = 10); **(c)** Bax and Bcl-2 expression of mice in each group (n = 10).

HE staining depicted (Fig. 2a) irregularly arranged and slightly swollen myocardial fibers without rupture in normal mice. MI/RI mice showed with dissolved, broken, or necrotic myocardial fibers, and increased interstitial and swelling. Treated with Sevo, MI/RI mice were exhibited with widened myocardial fiber space, reduced necrotic focus, and slightly swollen cardiomyocytes.

I/RI: ischemia/reperfusion injury; MI/RI: myocardial ischemia/reperfusion injury; Sevo: sevoflurane; HE: hematoxylin and eosin; TUNEL: transferase-mediated deoxyuridine triphosphate-biotin nick end labeling; *P < 0.05 compared with the sham group; #*P* < 0.05 compared with the I/R group; @Measurement data were expressed as mean ± standard deviation. One-way analysis of variance was utilized for discrepancy among groups, followed by Tukey’s multiple comparisons test.

As manifested by TUNEL staining and RT-qPCR assay (Figs. 2b and 2c), cardiomyocyte apoptosis was enhanced, which was evidenced by increased Bax and decreased Bcl-2 mRNA expression in MI/RI mice. After Sevo treatment, the alleviation was recognized in cardiac function, inflammation, oxidative stress, and cardiomyocyte apoptosis in MIRI mice. Overall, Sevo improves MI/RI in mice.

### Sevoflurane in combination with up-regulated miR-99a improves myocardial ischemia/reperfusion injury in mice

**Figure 3:**
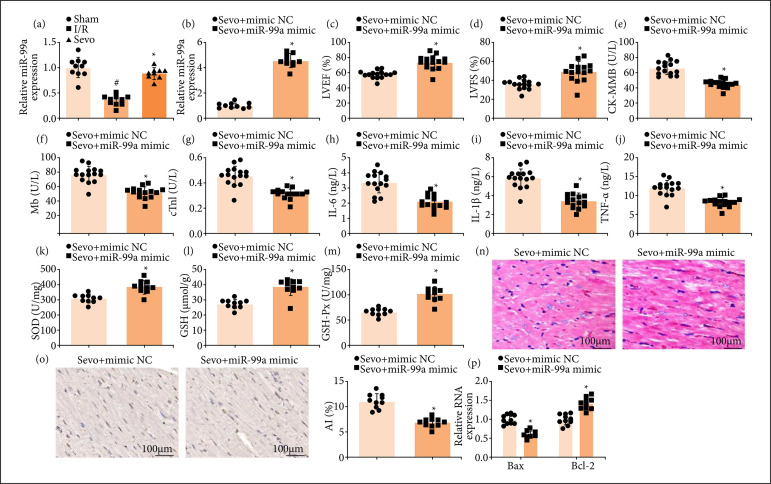
Sevo and up-regulated miR-99a in combination improves MI/RI in mice. (**a** and **b**) miR-99a expression of mice in each group (n = 10); (**c** and **d**) LVEF and LVFS of mice in each group (n = 15); (**e–g**) CK-MB, Mb, and cTnI contents in serum of mice in each group (n = 15); (**h–j**) IL-6, IL-1β, and TNF-α contents in serum of mice in each group (n = 15); (**k–m**) SOD, GSH, and GSH-Px levels of mice in each group (n = 10); **(n)** representative myocardial tissues stained by HE solution (n = 10); **(o)** representative myocardial tissues stained by TUNEL staining solution (n = 10); **(p)** Bax and Bcl-2 expression of mice in each group (n = 10).

MiR-99a has been reported to down-regulate in mice with cerebral I/R injury and plays a neuroprotective role^23^,^24^. Related experiments were performed to further explore whether Sevo affected myocardial oxidative stress and inflammatory response by up-regulating miR-99a. RT-qPCR detection revealed that miR-99a was down-regulated in myocardial tissues in MI/RI mice (Fig. 3a). Also, it was confirmed that Sevo elevated miR-99a expression in MI/RI mice, and this effect was further enhanced by miR-99a mimic treatment (Fig. 3b).

I/RI: ischemia/reperfusion injury; MI/RI: myocardial ischemia/reperfusion injury; Sevo: sevoflurane; LVEF: left ventricular ejection fraction; LVFS: left ventricular fractional shortening; CK-MB: creatine kinase-myocardial bound; Mb: myoglobin; cTnI: cardiac troponin I; IL: interleukin; TNF-α: tumor necrosis factor-α; SOD: superoxide dismutase; GSH: glutathione; GSH-Px: glutathione peroxidase; HE: hematoxylin and eosin; TUNEL: transferase-mediated deoxyuridine triphosphate-biotin nick end labeling; *P < 0.05 compared with the Sevo + mimic NC group; #*P* < 0.05 compared with the sham group; ^*P* < 0.05 compared with the I/R group; @measurement data were expressed as mean ± standard deviation. Discrepancy between two groups was assessed by independent sample t-test. One-way analysis of variance was utilized for discrepancy among groups, followed by Tukey’s multiple comparisons test.

Furthermore, it was detected that Sevo combined with miR-99a up-regulation functioned better to improve the cardiac function, reduce CK-MB, Mb, cTnI, IL-6, IL-1β, and TNF-α contents and reinforce SOD and GSH-Px activities and GSH content (Fig. 3c–3m).

Treated with Sevo and miR-99a up-regulation, MI/RI mice were exhibited with widened myocardial fiber space, reduced necrotic focus, and slightly swollen cardiomyocytes, as well as reduced apoptosis of cardiomyocytes (Fig. 3n-3p). To conclude, Sevo and up-regulated miR-99a in combination improves MI/RI in mice.

### miR-99a down-regulation reverses the effects of sevoflurane on myocardial ischemia/reperfusion injury mice

**Figure 4:**
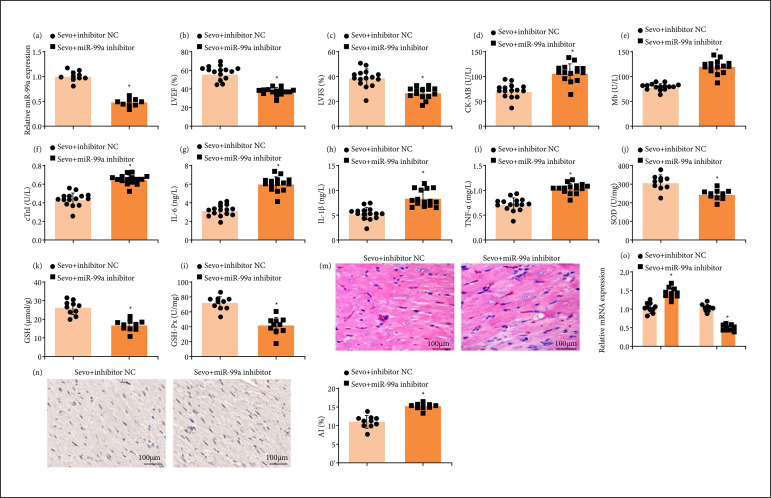
miR-99a down-regulation reverses the effects of Sevo on MI/RI mice. **(a)** miR-99a expression of mice in each group (n = 10); (**b** and **c**) LVEF and LVFS of mice in each group (n = 15); (**d**–**f**) CK-MB, Mb, and cTnI contents in serum of mice in each group (n = 15); (**g** and **i**) IL-6, IL-1β, and TNF-α contents in serum of mice in each group (n = 15); (**j** and **l**) SOD, GSH, and GSH-Px levels of mice in each group (n = 10); **(m)** representative myocardial tissues stained by HE solution (n = 10); **(n)** representative myocardial tissues stained by TUNEL staining solution (n = 10); **(o)** Bax and Bcl-2 expression of mice in each group (n = 10).

After miR-99a inhibitor treatment in MI/RI mice, it was determined that miR-99a inhibitor reversed Sevo-mediated promotion of miR-99a expression (Fig. 4a). Moreover, miR-99a down-regulation reversed the effects of Sevo on cardiac function, inflammation, oxidative stress, pathological damage, and apoptosis of cardiomyocytes of MI/RI mice. Shortly, miR-99a down-regulation reversed the effects of Sevo on MI/RI mice (Fig. 4b–4o).

MI/RI: myocardial ischemia/reperfusion injury; Sevo: sevoflurane; LVEF: left ventricular ejection fraction; LVFS: left ventricular fractional shortening; CK-MB: creatine kinase-myocardial bound; Mb: myoglobin; cTnI: cardiac troponin I; IL: interleukin; TNF-α: tumor necrosis factor-α; SOD: superoxide dismutase; GSH: glutathione; GSH-Px: glutathione peroxidase; HE: hematoxylin and eosin; TUNEL: transferase-mediated deoxyuridine triphosphate-biotin nick end labeling; *P < 0.05 compared with the Sevo + inhibitor NC group; #measurement data were expressed as mean ± standard deviation. Discrepancy between two groups was assessed by independent sample t-test.

### miR-99a targets bromodomain-containing protein 4

**Figure 5:**
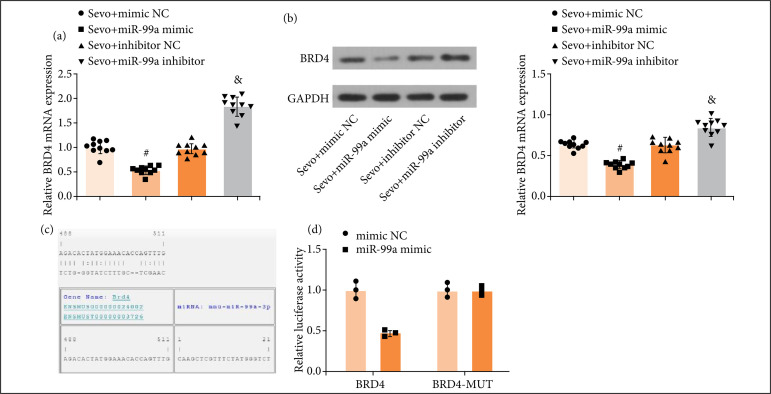
miR-99a targets BRD4. (**a** and **b**) BRD4 mRNA and protein expression of mice in each group (n = 10); **(c)** the targeting relationship between miR-99a and BRD4 predicted by bioinformatics software; **(d)** the targeting relationship between miR-99a and BRD4 validated by dual luciferase reporter gene assay.

RT-qPCR and Western blot assay revealed that miR-99a overexpression reduced BRD4 expression, and vice versus (Figs. 5a and 5b).

Online prediction by Jefferson suggested the existence of a specific binding region between miR-99a and BRD4 (Fig. 5c). Further validated by dual luciferase reporter gene assay, it was manifested that miR-99a mimic had no significant effect on the MUT-BRD4 plasmid luciferase activity, but impaired WT-BRD4 reporter plasmid luciferase activity (Fig. 5d).

BRD4: *bromodomain-containing protein 4;* GAPDH: glyceraldehyde-3-phosphate dehydrogenase; #*P* < 0.05 compared with the Sevo + mimic NC group; &*P* < 0.05 compared with the Sevo + inhibitor NC group; *P < 0.05 compared with the mimic NC group; @measurement data were expressed as mean ± standard deviation. Discrepancy between two groups was assessed by independent sample t-test. One-way analysis of variance was utilized for discrepancy among groups, followed by Tukey’s multiple comparisons test.

### Overexpression of bromodomain-containing protein 4 reverses up-regulation of miR-99a-induced attenuation of myocardial ischemia/reperfusion injury in mice

**Figure 6:**
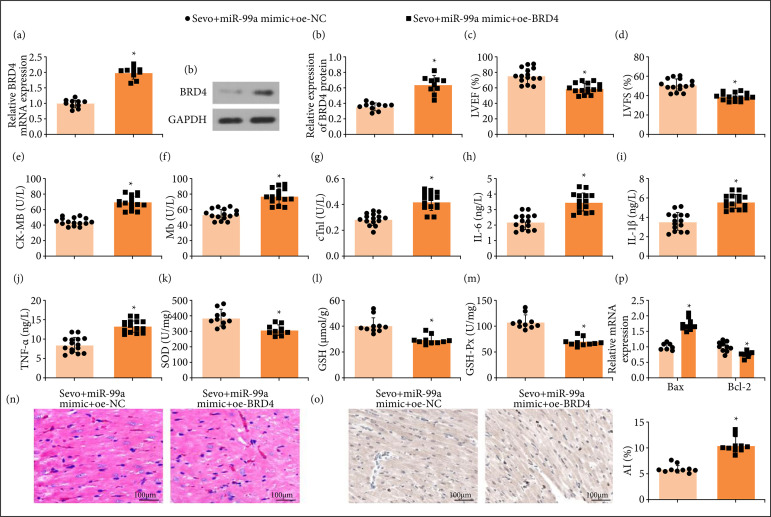
Overexpression of BRD4 reverses up-regulation of miR-99a-induced attenuation of MI/RI in mice. (a and b) BRD4 mRNA and protein expression of mice in each group (n = 10); (**c** and **d**) LVEF and LVFS of mice in each group (n = 15); (**e**–**g**) CK-MB, Mb, and cTnI contents in serum of mice in each group (n = 15); (**h**–**j**) IL-6, IL-1β, and TNF-α contents in serum of mice in each group (n = 15); (**k**–**m**) SOD, GSH, and GSH-Px levels of mice in each group (n = 10); **(n)** representative myocardial tissues stained by HE solution (n = 10); **(o)** representative myocardial tissues stained by TUNEL staining solution (n = 10); **(p)** Bax and Bcl-2 expression of mice in each group (n = 10).

As reported, BRD4 is up-regulated in AMI rats, and inhibiting BRD4 could reduce apoptosis of cardiomyocytes^17^. To probe the effect of BRD4 on the Sevo/miR-99a axis in MI/RI, oe-BRD4 was injected in MI/RI mice on the basis of Sevo and miR-99a mimic treatment. RT-qPCR and Western blot unveiled that oe-BRD4 elevated BRD4 expression, which was suppressed by Sevo and miR-99a mimic (Figs. 6a and 6b). Subsequently, it was observed that up-regulating BRD4 reversed the improving effect of up-regulating miR-99a on the cardiac function, inflammation, oxidative stress, pathological damage, and apoptosis of cardiomyocytes in MI/RI mice (Figs. 6c–6p).

BRD4: bromodomain-containing protein 4; GAPDH: glyceraldehyde-3-phosphate dehydrogenase; LVEF: left ventricular ejection fraction; LVFS: left ventricular fractional shortening; CK-MB: creatine kinase-myocardial bound; Mb: myoglobin; cTnI: cardiac troponin I; IL: interleukin; TNF-α: tumor necrosis factor-α; SOD: superoxide dismutase; GSH: glutathione; GSH-Px: glutathione peroxidase; MI/IR: myocardial ischemia/reperfusion injury; HE: hematoxylin and eosin; TUNEL: transferase-mediated deoxyuridine triphosphate-biotin nick end labeling; *P < 0.05 compared with the Sevo + miR-99a mimic + oe-NC group; #measurement data were expressed as mean ± standard deviation. Discrepancy between two groups was assessed by independent sample t-test.

## Discussion

MI is a leading cause of morbidity and mortality worldwide. With the increasing use of reperfusion therapy in patients with AMI, ischemia-induced MI is significantly reduced, but reperfusion-induced cardiac injury is increasingly apparent^25^. MI/RI is a complicated pathophysiological issue with the enhancement of inflammatory response, oxidative stress, and apoptosis^26^. Although studies have implied the substantial potentials of Sevo and miRNAs in this disease, the involvement of Sevo combined with miR-99a inMI/RI needs more comprehensive illustration. Given that, this study was launched, and the result highlighted that Sevo up-regulated miR-99a to protect against MI/RI through targeting BRD4.

At first, Sevo was examined to improve cardiac function, relieve myocardial injury, repress cardiomyocyte apoptosis, and alleviate inflammation and oxidative stress in mice with MI/RI. As discussed by a current research, Sevo post-conditioning narrows myocardial infarct size and improves cardiac function in MI/RI^8^. Supplementary to our findings, another study has elucidated that Sevo post-conditioning facilitates MI/RI improvement, which is reflected by ameliorated cardiac function (increased LVEF), smaller infarct size, and suppressed apoptosis^9^. Experimentally, the pathological status of Sevo-treated MI/RI mice is ameliorated, which is witnessed by promoted cardiac function, decreased infarct size, and repressed cardiomyocyte apoptosis^27^.

Supportive to the findings in this study, Sevo exposure is documented to be capable of attenuating cardiac insufficiency, disturbing cardiac infarction, reducing infarction area, and inhibiting oxidative stress^28^. In addition, there is an observational study highlighting that Sevo improves cardiac function and hemodynamics, promotes pathological damage of myocardial tissues and cardiomyocytes ultrastructure, reduces myocardial infarction area, and cardiomyocyte apoptosis by up-regulating miR-145^29^. The aforesaid studies confirmed the protective roles of Sevo in heart-related diseases.

Next, miR-99a expression was investigated to be down-regulated in myocardial tissues, and BRD4 was up-regulated in myocardial tissues in MI/RI mice. In fact, it has been previously studied that miR-99a is down-regulated in cerebral I/R injury patients, and its overexpression attenuates I/R injury, inhibits neuronal apoptosis, reinforces cell viability, and suppresses hydrogen peroxide-induced oxidative stress^23^. Moreover, it is confirmed that miR-99a up-regulation alleviates brain tissue damage and suppresses apoptosis in cerebral I/R injury^24^.

Besides that, there is a study recording a reduction in miR-99a expression in patients with AMI^14^. Also, it has been elucidated that miR-99a expression is decreased in MI, and miR-99a enhancement improves cardiac function and raises survival rate of mice with MI by impeding apoptosis and encouraging autophagy^15^. Suggested by an academic research, miR-99a expression trends toward a reduction in H9C2 cells in hypoxia-induced injury, and silencing miR-99a functions a suppressor for cell viability and a activator for cell apoptosis in myocardial infarction^30^.

Additionally, it is surveyed that miR-99a restoration hinders oxidative stress and apoptosis of cardiomyocytes in lipopolysaccharide-induced oxidative injury^13^. In terms of BRD4, there is a study revealing that it is overexpressed in renal I/RI, and BRD4 depletion impedes cell apoptosis and oxidative stress^31^. Intriguingly, incremental BRD4 protein expression is recognized in cardiomyocytes in myocardial infarction, and its knockdown attenuates cardiomyocyte apoptosis^17^. Also, BRD4 expression is elevated in AMI rat models and BET down-regulation reverses cardiac function injury, and reduces serum CK-MB and IL-6 contents^32^.

In the present work, BRD4 overexpression was tested to reverse the improving effects of miR-99a on MI/RI mice. It has been validated that suppression of BRD4 retards the process of heart failure^33^. Moreover, another paper has highlighted that up-regulating BRD4 in diabetic cardiomyopathy causes mitochondrial damage and impairs cardiac function, while down-regulating BRD4 has the opposite effect^34^. In addition to that, it is known that depletion of BRD4 reduces fibrosis, inflammatory and oxidative stress reactions in cardiac hypertrophy^35^.

Based on the finding of this article, it was found that miR-99a targeted BRD4. Besides, up-regulating BRD4 reversed the improving effect of up-regulating miR-99a on the cardiac function, inflammation, oxidative stress, pathological damage, and apoptosis of cardiomyocytes in MI/RI mice, suggesting that the miR-99a/BRD4 axis functions in MI/RI. However, the mechanisms of the miRNA/BRD4 axis in MI/RI need further investigation.

## Conclusion

In summary, the functional roles of Sevo, miR-99a, and BRD4 in MI/RI have been concluded as Sevo and up-regulated miR-99a attenuate MI/RI through inhibiting BRD4. The present study provides a new target for the cardiac function of sevoflurane in myocardial tissues of MI/RI and for the treatment of cardiovascular I/R injury. However, since there is an inversely proportional relationship between the expression of miR-99a and BDR4, the protection mechanism of the miR-99a/BRD4 axis in MI/RI should be further explored.

## Data Availability

The data will be available upon request.

## Supplementary Table 1

**Supplementary Table 1:** Primer sequences for genes used in our study.

Gene	Primer sequence
miR-99a	Forward: 5’-AACCCGTAGATCCGATCTTGTG-3’
	Reverse: 5’-CACAAGATCGGATCTACGGGTT-3’
U6	Forward: 5’-CTCGCTTCGGCAGCACATA-3’
	Reverse: 5’-AACGATTCACGAATTTGCGT-3’
BRD4	Forward: 5’-CCTCCCAAATGTCTACAACGC-3
	Reverse: 5’-TGAGCAGATATTGCAGTTGGTT-3’
Bax	Forward: 5’-CCCGAGAGGTCTTTTTCCGAG-3
	Reverse: 5’-CCAGCCCATGATGGTTCTGAT-3’
Bcl-2	Forward: 5’-TGAGGACCCAATCTGGAAACC-3
	Reverse: 5’-AAACCCAAACAAATACATAAGGCAA-3’
GAPDH	Forward: 5’-AGGTCGGTGTGAACGGATTTG-3’
	Reverse: 5’-TGTAGACCATGTAGTTGAGGTCA-3’

miR-99a: microRNA-99a; BRD4: Bromodomain-containing protein 4; GAPDH: glyceraldehyde-3-phosphate dehydrogenase.
